# Assessment of ultra-pulse CO_2_ laser therapy in comparison to sequential laser and drug treatments for scar reduction

**DOI:** 10.1097/MD.0000000000041819

**Published:** 2025-03-14

**Authors:** Xiang-Jun Chen, Di Wu, Shu-Xia Kang, Tian-Jiao Xing, Yao Yao, Li Yu, Jun-Qing Liang

**Affiliations:** a Department of Plastic and Reconstructive Surgery, Peking University Cancer Hospital Inner Mongolia Hospital, Hohhot, China; b Department of Dermatology and Venereology, Peking University Cancer Hospital Inner Mongolia Hospital, Hohhot, China; c Department of Intensive Care Medicine, PLA Joint Logistics Support Force 969th Hospital, Hohhot, China; d Breast Tumor Center, Peking University Cancer Hospital Inner Mongolia Hospital, Hohhot, China.

**Keywords:** proliferative burn scars, scar reduction, sequential laser therapy, ultra-pulse CO_2_ laser therapy, Vancouver Scar Scale

## Abstract

Scar management, particularly for early proliferative burn scars, remains a clinical challenge. This study assesses the efficacy of ultra-pulse carbon dioxide (CO_2_) laser therapy in comparison to sequential laser therapy and pharmacological interventions for scar reduction. A retrospective evaluation was conducted from January 2016 to March 2019 involving 200 patients with early proliferative burn scars treated at the Burn and Plastic Surgery Department of our institution. Participants were assigned to 4 groups: Group A received ultra-pulse CO_2_ laser therapy, Group B underwent sequential pulsed dye laser therapy, Group C received sequential laser therapy combined with pharmacological treatment, and a control group received no intervention. Clinical outcomes were assessed using the Vancouver Scar Scale (VSS) and the Numeric Pain Rating Scale. Efficacy was evaluated based on scar characteristics and pain scores. Demographic characteristics across all groups were comparable, with no significant differences noted (*P* > .05). The clinical efficacy assessment revealed that the overall effective rates for Group A, Group B, and Group C were 80.00%, 96.00%, and 98.00%, respectively. Groups B and C not only exhibited significantly higher effective rates but also demonstrated marked improvements in scar characteristics as measured by the VSS, including reduced erythema and thickness. Additionally, pain scores during treatment were lowest in Group C, indicating better tolerability compared to the other modalities (*P* < .05). Sequential laser therapy improves the clinical efficacy for early proliferative burn scars, enhancing scar characteristics overall. When combined with pretreatment pharmacotherapy, this approach also reduces patient pain during treatment. These results highlight the benefits of integrating sequential laser and drug therapies in scar management.

## 1. Introduction

Scarring is a common dermatological concern that can significantly impact an individual’s quality of life. Scars may arise from various etiologies, including surgical interventions, trauma, or pathological skin conditions, leading to both physical and psychological burdens. The pursuit of effective treatments for scar reduction has prompted the exploration of several modalities, with laser therapy emerging as a prominent option due to its targeted action and minimal invasiveness.^[1–3]^ Ultra-pulse carbon dioxide (CO_2_) laser therapy represents a sophisticated approach within the spectrum of laser treatments. This modality utilizes a specific wavelength (10,600 nm) to penetrate the epidermis and reach the dermal layer, promoting collagen remodeling while minimizing damage to surrounding tissues. The unique capability of ultra-pulse CO_2_ lasers to deliver energy in a fractionated manner allows for enhanced healing and reduced downtime, making it a desirable choice for managing various types of scars, including hypertrophic scars and keloids.^[4,5]^

In contrast, sequential laser treatments, such as pulsed dye laser (PDL) therapy, have also gained attention for their efficacy in scar management. PDL operates at a wavelength of 595 nm and targets vascular lesions within the scar tissue, promoting improved color and texture. The sequential approach combines different laser modalities to optimize treatment outcomes, as evidenced by protocols that initially utilize PDL to address vascularity followed by ultra-pulse CO_2_ laser therapy for deeper tissue remodeling.^[6,7]^ Moreover, the integration of pharmacological agents in conjunction with laser therapies has shown promise in enhancing treatment effectiveness. For instance, the administration of local anesthetics and anti-inflammatory medications prior to laser treatment may augment patient comfort and potentially improve therapeutic outcomes. Specifically, formulations containing lidocaine and corticosteroids can be employed to manage pain and inflammation, facilitating a more tolerable treatment experience and possibly enhancing the regenerative effects of laser therapy.^[8,9]^

The objective of this study is to assess the comparative effectiveness of ultra-pulse CO_2_ laser therapy against sequential laser and drug treatments in reducing scars. This study will contribute to the existing body of knowledge by providing insights into the efficacy of ultra-pulse CO_2_ laser therapy in comparison to sequential laser treatments and pharmacological interventions, ultimately enhancing clinical practice and patient care in dermatological settings.

## 2. Methods

### 2.1. Study design

This study involved a retrospective evaluation conducted at our hospital to assess the effectiveness of Ultra-Pulse CO_2_ laser therapy in comparison to sequential laser and pharmacological treatments for scar reduction. The research spanned from January 2016 to March 2019 and included 200 patients with early proliferative burn scars, defined as scars occurring within 1 month post-wound healing, treated at the Burn and Plastic Surgery Department. Participants were assigned to 1 of the 4 groups using a random number table: Group A (n = 50), Group B (n = 50), Group C (n = 50), and a control group (n = 50), based on the treatment they received. The research methodology, objectives, and protocols were developed in accordance with the STROBE (Strengthening the Reporting of Observational Studies in Epidemiology) guidelines^[10]^ and received approval from the Ethics Committee of our hospital (2024-KY0605-131). Informed consent was obtained from the patient for publication of this case report details.

### 2.2. Inclusion and exclusion criteria

Inclusion criteria:

1. Diagnosis of proliferative scars: participants must be clinically confirmed to have early proliferative burn scars resulting from thermal injuries, with a scar surface area not exceeding 3% of the total body surface area.
2. Age requirement: patients must be over 16 years of age to ensure sufficient maturity for informed consent and understanding of the study procedures.
3. Cognitive status: subjects must be alert and oriented, capable of providing informed consent and participating in the study protocol.

Exclusion criteria:

1. Allergic reactions: individuals with a documented history of hypersensitivity or allergic reactions to corticosteroids, triamcinolone acetonide, 5-fluorouracil, or other related medications will be excluded to avoid potential adverse reactions during treatment.
2. Mental health conditions: patients with diagnosed psychiatric disorders or significant cognitive impairments that could affect their ability to understand or comply with the study requirements will not be eligible.
3. Concurrent injuries: participants with additional traumatic injuries or burn scars that may complicate the treatment process or confound the results will be excluded from the study.
4. Loss to follow-up: patients who are lost to follow-up during the study period or who withdraw consent will be excluded from the final analysis to maintain the integrity of the data collected.

### 2.3. Treatment protocols

Group A: Patients in this group underwent ultra-pulse CO_2_ fractional laser (UPCL) therapy, utilizing the UltraPulse Encor CO_2_ laser device acquired from Lumenis, USA. The laser operates at a wavelength of 10,600 nm and offers 3 selectable treatment modes: Active FX, Deep FX, and SCAAR FX. Prior to treatment, a compounded lidocaine cream was applied topically to the scar areas to ensure surface anesthesia, which was sealed with a transparent plastic film for 1 hour before being washed off. The selection of the treatment mode and parameters was determined based on scar thickness: for scars <1 mm thick, the Deep FX mode was used with an energy setting of 25 to 50 mJ and a treatment density of 5% to 10%. For scars measuring 1 mm or greater, the SCAAR FX mode was applied, utilizing energy levels of 80 to 150 mJ and a treatment density of 3% to 5%. Treatment sessions were scheduled every 3 months.

Group B: This group received sequential laser therapy, beginning with PDL treatment. Similar to Group A, a compounded lidocaine cream was applied to the scar areas for surface anesthesia, covered with a plastic film for 1 hour before removal. The Vbeam Platinum PDL device, also from Candela, USA, was employed, operating at a wavelength of 595 nm. Treatment commenced with a low energy test, followed by adjustments based on patient response. Parameters included a spot size ranging from 7 to 12 mm, pulse widths of 1.5 to 3.0 ms, and energy densities of 8.0 to 9.5 J/cm^2^, ensuring minimal overlap in treatment areas. Sessions were conducted monthly, and once the degree of scar erythema decreased to mild levels, patients transitioned to UPCL therapy, following the same methodology as Group A.

**Figure 1.**
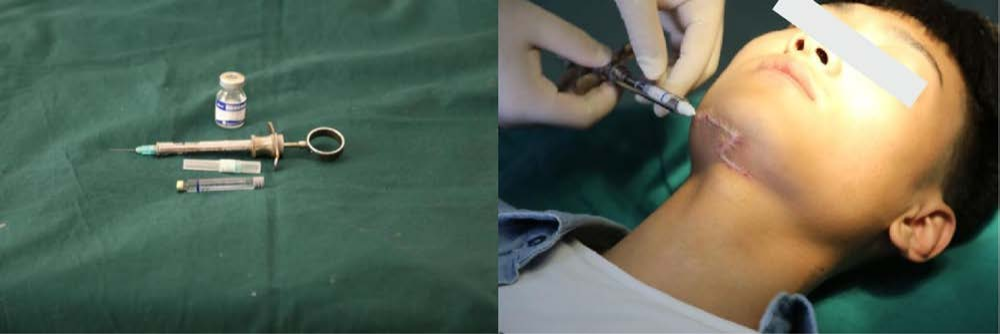
A dental local anesthesia syringe was used for easier injection in cases of high scar rigidity.

Group C: Patients in this group received sequential laser therapy combined with pharmacological intervention. The initial laser treatment protocol was identical to that of Group B. However, prior to the laser application, a drug mixture composed of 2% lidocaine, triamcinolone acetonide, and 5-fluorouracil was administered approximately 30 minutes before the laser treatment. The drug mixture was prepared in a ratio of 5:1:0.6, with the dosage determined by the scar area of each patient. A disposable luer lock syringe was used to inject the mixture slowly and evenly into the entirety of the hypertrophic scar. In cases of high scar rigidity, a dental local anesthesia syringe was utilized for easier injection (Fig. 1).

Following laser treatment, all patients were instructed to apply ice packs to the treated areas, which were maintained for a specified duration. The total treatment and observation period for all groups extended for 12 months. Control Group: The control group did not receive any intervention but was monitored for 12 months to assess the natural progression of the scars without treatment.

### 2.4. Clinical outcomes and assessment criteria

The study evaluated various clinical outcomes across the 3 treatment groups (A, B, and C), as well as a control group. The following indicators were assessed:

Clinical efficacy: the primary focus was to determine the overall clinical effectiveness of the treatments administered in each group, considering both subjective and objective measures.

Pain scores during procedures: pain experienced during treatment was quantified using the Numeric Pain Rating Scale, where participants rated their pain on a scale from 0 to 10. A higher score indicated greater pain intensity experienced during the procedure.

In addition to the above measures, further comparisons were made among the 3 treatment groups (A, B, and C) and the control group based on the following criteria:

Scar assessment: the Vancouver Scar Scale (VSS) was employed to evaluate scar characteristics, including erythema, thickness, and pliability. Each parameter was scored on a scale from 0 to 5, where higher scores corresponded to more severe clinical manifestations: A score of 0 indicates no erythema, with thickness and pliability equivalent to normal skin. A score of 1 represents mild erythema, a thickness increase of less than or equal to 1 mm, and pliability changes only under minimal pressure. A score of 2 denotes moderate erythema, with a thickness increase of up to 2 mm and pliability changes under moderate pressure. A score of 3 reflects moderate erythema, with a thickness increase of up to 3 mm and significant resistance to deformation that requires substantial pressure. A score of 4 indicates severe erythema (where the scar appears red), a thickness increase of up to 4 mm, and an inability to deform. Finally, a score of 5 represents very severe erythema (where the scar appears purple), a thickness increase >4 mm, and significant contracture.

Itch scores: the severity of itch associated with the scars was assessed using a Visual Analog Scale, which ranges from 0 to 10. Higher scores were directly proportional to the intensity of pruritus experienced by the patients.

### 2.5. Efficacy evaluation criteria

The efficacy of the treatments was assessed using specific criteria categorized into 3 classifications, with the following detailed evaluations:

1. Marked improvement: defined by the complete disappearance of erythema associated with the scar and a scar thickness that is essentially equivalent to that of normal skin. The erythema was visually assessed using the VSS, with standardized photographs taken at multiple angles and lighting conditions to ensure consistency. Scar thickness was measured using digital calipers.
2. Effective improvement: indicated a significant reduction in erythema severity, with a decrease in scar thickness of at least 50%. Erythema reduction was also scored using the VSS, and scar thickness was assessed through digital calipers or imaging at the same time points. A ≥ 50% decrease in scar thickness was confirmed by 2 independent evaluators who were blinded to the treatment group. Additionally, photographs were taken for consistency in visual assessment, and the measurements were averaged between the evaluators. Discrepancies in evaluation were resolved through a consensus approach.
3. Ineffective: cases classified as ineffective showed no observable reduction in erythema severity and a decrease in scar thickness of <50%. A lack of improvement in erythema or thickness was confirmed through standardized photography and caliper measurements taken at each time point. The final assessment of effectiveness was based on the average of 3 evaluators to minimize evaluator bias.

The overall effective rate was calculated using the formula: total effective rate = (number of marked improvements + number of effective improvements)/total number of cases × 100%. The overall efficacy was assessed by comparing pre- and posttreatment measurements of erythema, thickness, and pliability at each time point. Statistical significance was calculated using paired t-tests for continuous variables and Chi-square tests for categorical data.

To ensure consistency across all evaluators, we implemented a training session before the study to standardize assessment procedures, including the use of the VSS and calipers. Additionally, inter-rater reliability was evaluated, with a consistency threshold set at >80% for measurement concordance. This comprehensive approach to evaluating treatment efficacy provides a robust and quantifiable assessment of therapeutic outcomes, ensuring that clinical decisions are based on reliable and reproducible data.

### 2.6. Statistical analysis

Statistical analyses were conducted with precision using SPSS software (Version 27.0). Quantitative data that adhered to a normal distribution were assessed for inter-group differences employing independent sample *t*-tests, with results expressed as mean ± standard deviation. For categorical data, frequencies and percentages were utilized for representation, and the independence or associations among these variables were examined through Chi-square (χ^2^) tests. In instances where the conditions for Chi-square tests were not met, Fisher exact probability method was employed to ensure accurate statistical evaluation. All hypotheses were tested using a 2-tailed approach, and a significance level was established at a *P*-value of <.05 to determine statistical significance.

## 3. Results

### 3.1. Demographic characteristics of study groups

In Group A, participants ranged in age from 22 to 56 years, with a mean age of 38.35 ± 10.59 years, comprising 18 males and 32 females. Group B included individuals aged 21 to 57 years, with an average age of 38.16 ± 10.63 years, consisting of 17 males and 33 females. Group C featured participants aged 21 to 58 years, yielding a mean age of 38.49 ± 10.24 years, with 16 males and 34 females. The control group encompassed subjects aged 20 to 57 years, with a mean age of 38.02 ± 10.31 years, including 15 males and 35 females. Statistical comparisons of demographic data among the groups revealed no significant differences, as indicated by *P*-values >.05, thereby affirming the comparability of the study.

### 3.2. Results of clinical efficacy assessment

The clinical efficacy of the treatments was evaluated across 3 groups: Group A (ultra-pulse CO_2_ laser therapy), Group B (sequential PDL therapy), and Group C (combined sequential laser and pharmacotherapy). The overall effective rates were significantly higher in Groups B and C compared to Group A. Specifically, the overall effective rate for Group A was 80.00%, while Groups B and C demonstrated rates of 96.00% and 98.00%, respectively. Statistical analysis revealed that the differences between Group A and both Groups B and C were statistically significant (*P* < .05). However, there was no significant difference in efficacy observed between Groups B and C (*P* > .05; Table 1). These findings indicate that both the Sequential PDL Therapy and the combined sequential laser and pharmacotherapy approaches yielded superior clinical outcomes compared to the ultra-pulse CO_2_ laser therapy. The substantial improvement in clinical efficacy in the latter 2 groups suggests that incorporating pharmacotherapy with laser treatment may enhance therapeutic results for patients.

**Table 1:** Comparison of clinical efficacy among treatment groups (n [%]).

Group	Number of cases (n)	Marked improvement (n, %)	Improvement (n, %)	No improvement (n, %)	Overall effective rate (n, %)
Group A	50	23 (46.00)	17 (34.00)	10 (20.00)	40 (80.00)
Group B	50	29 (58.00)	19 (38.00)	2 (4.00)	48 (96.00)*
Group C	50	32 (64.00)	17 (34.00)	1 (2.00)	49 (98.00)*

Group A: Ultra-Pulse CO_2_ Laser Therapy Group

Group B: Sequential Pulsed Dye Laser Therapy Group

Group C: Combined Sequential Laser and Pharmacotherapy Group

* Statistical significance: *P* < .05 when compared to Group A.

### 3.3. Posttreatment analysis of scar characteristics across treatment groups

The assessment of scar characteristics, including erythema, thickness, and pliability scores, demonstrated significant reductions following treatment in all 3 groups (*P* < .05). Notably, the posttreatment scores for erythema, thickness, and pliability in Groups B and C were significantly lower than those in Group A (*P* < .05), while no significant difference was observed between Groups B and C (*P* > .05). Furthermore, when comparing pretreatment scores, there were no significant differences between Groups A, B, and C and the control group (*P* > .05). However, the posttreatment scar scores for all 3 treatment groups were significantly lower than those of the control group (*P* < .05; Table 2). These findings highlight the effectiveness of the treatment modalities employed in reducing scar severity compared to the control group.

**Table 2:** Comparative analysis of scar scores before and after treatment among different groups (mean ± SD).

Group	Time	Erythema score (mean ± SD)	Thickness score (mean ± SD)	Pliability score (mean ± SD)
Group A (n = 50)	Pretreatment	3.04 ± 0.61	3.15 ± 0.62	2.72 ± 0.54
	Posttreatment	2.43 ± 0.52*,†	2.52 ± 0.54*,†	2.19 ± 0.46*,†
Group B (n = 50)	Pretreatment	3.02 ± 0.64	3.13 ± 0.63	2.71 ± 0.56
	Posttreatment	1.91 ± 0.47*,†,‡	1.98 ± 0.50*,†,‡	1.73 ± 0.42*,†,‡
Group C (n = 50)	Pretreatment	3.01 ± 0.62	3.10 ± 0.65	2.69 ± 0.58
	Posttreatment	1.87 ± 0.45*,†,‡	1.94 ± 0.51*,†^,^‡	1.70 ± 0.41*,†,‡
Control group (n = 50)	–	3.03 ± 0.57	3.12 ± 0.56	2.70 ± 0.49

Group A: Ultra-Pulse CO_2_ Laser Therapy Group.

Group B: Sequential Pulsed Dye Laser Therapy Group.

Group C: Combined Sequential Laser and Pharmacotherapy Group.

* Statistical significance: *P* < .05 compared to pretreatment scores within the same group.

† Statistical significance: *P* < .05 compared to control group scores.

‡ Statistical significance: *P* < .05 compared to Group A posttreatment.

### 3.4. Evaluation of pruritus scores pre- and posttreatment across groups

The pruritus scores across all 3 treatment groups exhibited a significant decrease following treatment (*P* < .05). Specifically, the posttreatment pruritus scores in Groups B and C were significantly lower than those in Group A (*P* < .05), while no significant difference was observed between Groups B and C (*P* > .05). In terms of pretreatment assessments, the pruritus scores for Groups A, B, and C did not differ significantly from those of the control group (*P* > .05). However, posttreatment pruritus scores for all 3 treatment groups were significantly lower than those of the control group (*P* < .05; Table 3). These results underscore the effectiveness of the treatment modalities in alleviating pruritus compared to the control condition.

**Table 3:** Comparative analysis of pruritus scores before and after treatment among different groups (mean ± SD).

Group	Time	Pruritus score (mean ± SD)
Group A (n = 50)	Pretreatment	5.62 ± 1.43
	Posttreatment	4.19 ± 1.08*,†
Group B (n = 50)	Pretreatment	5.41 ± 1.48
	Posttreatment	3.10 ± 0.95*,†,‡
Group C (n = 50)	Pretreatment	5.49 ± 1.46
	Posttreatment	3.05 ± 0.92*,†,‡
Control group (n = 50)	–	5.52 ± 1.37

Group A: Ultra-Pulse CO_2_ Laser Therapy Group.

Group B: Sequential Pulsed Dye Laser Therapy Group.

Group C: Combined Sequential Laser and Pharmacotherapy Group.

* Statistical significance: *P* < .05 compared to pretreatment scores within the same group.

† Statistical significance: *P* < .05 compared to control group scores.

‡ Statistical significance: *P* < .05 compared to Group A posttreatment.

### 3.5. Clinical outcomes of sequential laser therapy in scarring management

**Figure 2.**
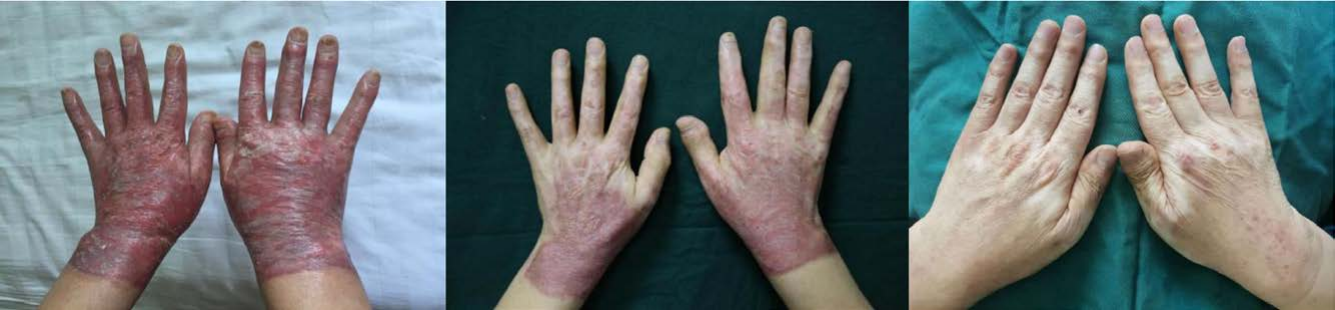
Photographic series showing the scar condition from left to right: before treatment, after 2 sessions of pulsed dye laser therapy, and after 4 sessions of fractional laser therapy.

**Figure 3.**
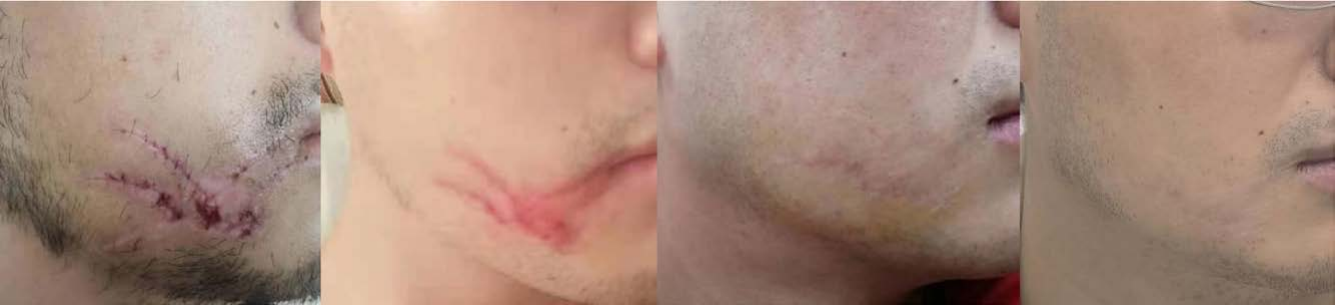
Photographic series illustrating the scar progression from left to right: before treatment, after 1 session of pulsed dye laser (PDL) therapy, after 4 sessions of PDL therapy, and after 2 sessions of fractional laser therapy. PDL = pulsed dye laser.

In our study, we observed significant improvements in scar appearance following the administration of combined treatment modalities involving PDL and UPCL therapy, along with pharmacological sequential therapy in patients with hypertrophic scars. A notable case involved a 51-year-old male patient who presented with hypertrophic scarring on both hands, which had developed 3 weeks post-burn injury. After undergoing the combined laser treatment regimen, there was a marked improvement in the scar’s characteristics, suggesting a positive response to the intervention (Fig. 2). The reduction in erythema and overall texture of the scar indicated enhanced healing and remodeling, which were visually apparent compared to the pretreatment assessment. Similarly, a 26-year-old male patient with post-surgical hypertrophic scarring on the right side of the face following trauma suturing also demonstrated significant improvement after receiving the same treatment combination (Fig. 3). The synergistic effect of the laser therapies, complemented by pharmacological support, contributed to a notable reduction in scar prominence and improved pliability, further confirming the efficacy of this integrated approach.

## 4. Discussion

Burn injuries are common traumatic events, primarily caused by exposure to high-temperature objects, electrical currents, or corrosive chemicals, leading to skin and soft tissue damage.^[11,12]^ While the initial treatment of burn wounds typically results in gradual healing, patients often develop pathological scars, specifically hypertrophic scars, which can persist for months or even years following injury.^[8–10]^ The resulting scar tissue is often characterized by poor elasticity, which compromises the structural integrity and functional capacity of the skin, potentially leading to functional impairments and associated symptoms such as pruritus. This not only diminishes the quality of life but can also contribute to psychological distress, including feelings of inadequacy and depression. Moreover, hypertrophic scars may exhibit inflammatory factor infiltration, increasing the risk of ulcer formation and, in rare cases, malignant transformation.^[13–15]^ Consequently, it is imperative to manage these scars proactively to improve patient outcomes.

Currently, treatment modalities for hypertrophic scars following burns include pharmacological therapy, surgical intervention, and laser therapy. Pharmacological treatments primarily involve topical applications aimed at restoring skin elasticity; however, these approaches often have prolonged treatment durations and may not effectively suppress scar proliferation. Surgical options, while potentially effective, carry risks of additional trauma and higher costs, making them less favorable as first-line treatments for hypertrophic scars.^[16,17]^ Laser therapy has gained recognition in scar treatment, with significant advancements in technology enhancing its application in clinical practice. Among various laser treatments, PDL and UPCL are commonly employed for managing hypertrophic scars. PDL targets hemoglobin selectively, promoting capillary closure and inhibiting scar tissue growth.^[18]^ In contrast, UPCL utilizes thermal effects to create localized zones of microheating on the scar surface, facilitating vascular disruption and vaporization of the scar tissue. This technique not only improves scar texture but also stimulates the reorganization of dermal collagen and elastic fibers, resulting in a smoother and more elastic skin surface.^[19,20]^

While previous studies have explored the use of PDL and UPCL individually or in combination, there is a notable lack of research on their sequential application. Our study innovatively utilized the distinct properties and penetration depths of both laser therapies in a sequential manner: initial PDL treatment was administered, followed by UPCL once significant erythema reduction was achieved.^[21,22]^ The results indicated that the scar and pruritus scores in both treatment groups (B and C) were significantly lower than those in the control group, with overall clinical efficacy rates in groups B and C exceeding that of group A (*P* < .05). These findings underscore the effectiveness of the sequential application of PDL and UPCL in alleviating hypertrophic scar symptoms and inhibiting scar proliferation. Additionally, in group C, patients received local injections of lidocaine, triamcinolone, and 5-fluorouracil prior to laser treatment. The use of lidocaine helped minimize procedural discomfort, while triamcinolone and 5-fluorouracil facilitated scar flattening and reduction, thereby enhancing the effectiveness of subsequent laser therapies. Notably, the clinical efficacy rates and incidence of adverse reactions in group C did not differ significantly from those in group B, although pain scores during the procedure were lower in group C. This suggests that pretreatment injections can effectively reduce procedural pain without compromising the outcomes of laser therapy.

This study acknowledges several limitations that warrant consideration. Firstly, the sample size, while sufficient for preliminary findings, may not capture the full variability of hypertrophic scar responses across diverse patient populations. Secondly, the follow-up duration was relatively short, limiting insights into the long-term efficacy and potential recurrence of scars posttreatment. Additionally, the absence of a standardized assessment tool for scar evaluation could introduce subjectivity in scoring. Future research should aim to include larger, multi-center trials with extended follow-up periods and standardized evaluation methods to further validate these findings and explore additional adjunctive therapies for enhanced scar management.

## 5. Conclusions

Sequential laser therapy significantly enhances the clinical efficacy for early proliferative burn scars, leading to comprehensive improvements in scar characteristics. Notably, when combined with pretreatment pharmacotherapy, this approach not only optimizes scar outcomes but also alleviates patient discomfort during treatment sessions. These findings underscore the value of integrating sequential laser techniques with pharmacological interventions in the management of burn scars.

## Acknowledgments

We appreciate the cooperation and informed consent provided by the patients for this study.

## Author contributions

**Conceptualization:** Xiang-Jun Chen, Di Wu, Yao Yao, Jun-Qing Liang.

**Data curation:** Di Wu, Shu-Xia Kang, Yao Yao.

**Formal analysis:** Xiang-Jun Chen, Shu-Xia Kang, Yao Yao.

**Investigation:** Xiang-Jun Chen, Di Wu, Shu-Xia Kang, Tian-Jiao Xing, Yao Yao.

**Methodology:** Di Wu, Tian-Jiao Xing, Li Yu, Jun-Qing Liang.

**Resources:** Xiang-Jun Chen, Di Wu, Shu-Xia Kang, Tian-Jiao Xing, Jun-Qing Liang.

**Supervision:** Tian-Jiao Xing.

**Writing – review & editing:** Li Yu.

**Writing – original draft:** Xiang-Jun Chen.
